# General practitioners’ approaches to prostate-specific antigen testing in the north-east of the Netherlands

**DOI:** 10.1186/s12875-020-01350-3

**Published:** 2020-12-17

**Authors:** Sanny Kappen, Lisa Koops, Verena Jürgens, Michael H. Freitag, Marco H. Blanker, Antje Timmer, Geertruida H. de Bock

**Affiliations:** 1 Division of Epidemiology and Biometry, Department of Health Services Research, Faculty of Medicine and Health Sciences, Carl von Ossietzky University Oldenburg, Oldenburg, Germany; 2 Department of General Practice and Elderly Care Medicine, University Medical Center Groningen, University of Groningen, Groningen, The Netherlands; 3 Division of General Practice, Department of Health Services Research, Faculty of Medicine and Health Sciences, Carl von Ossietzky University Oldenburg, Oldenburg, Germany; 4 Department of Epidemiology, University Medical Center Groningen, University of Groningen, Groningen, The Netherlands

**Keywords:** Prostatic neoplasms, Early detection of Cancer, Prostate-specific antigen, General practitioners, Physicians, Health care surveys, Guideline, Guideline adherence, Netherlands

## Abstract

**Background:**

There is wide variation in clinical practice for the early detection of prostate cancer, not least because of the ongoing debate about the benefits of prostate-specific antigen (PSA) testing. In this study, we aimed to assess the approaches, attitudes, and knowledge of general practitioners (GPs) regarding PSA testing in primary care in the Netherlands, particularly regarding recommendations for prostate cancer.

**Methods:**

Questionnaire surveys were sent to 179 GPs in the north-east of the Netherlands, of which 65 (36%) were completed and returned. We also surveyed 23 GPs attending a postgraduate train-the-trainer day (100%). In addition to demographic data and practice characteristics, the 31-item questionnaire covered the attitudes, clinical practice, adherence to PSA screening recommendations, and knowledge concerning the recommendations for prostate cancer early detection. Statistical analysis was limited to the descriptive level.

**Results:**

Most GPs (95%; *n* = 82) stated that they had at least read the Dutch GP guideline, but just half (50%; *n* = 43) also stated that they knew the content. Almost half (46%; *n* = 39) stated they would offer detailed counseling before ordering a PSA test to an asymptomatic man requesting a test. Overall, prostate cancer screening was reported to be of minor importance compared to other types of cancer screening.

**Conclusions:**

Clinical PSA testing in primary care in this region of the Netherlands seems generally to be consistent with the relevant guideline for Dutch GPs that is restrictive to PSA testing. The next step will be to further evaluate the effects of the several PSA testing strategies.

## Background

Prostate cancer is the second most frequent cancer and the fifth leading cause of cancer death in men worldwide [1, 2]. This estimated that there would be about 1.3 million new prostate cancer cases and 359,000 related deaths worldwide in 2018 [1, 2]. However, although the incidence of prostate cancer rose rapidly in most countries due to an increase in screening for prostate cancer by prostate-specific antigen (PSA) testing in the 1990s, it has been decreasing in the Netherlands over recent years [3–5]. In contrast, the country-specific standardized PCa mortality showed a steady decrease from 35.1 per 100,000 in 1995 to 22.2 per 100,000 in 2016 [4].

Prostate cancer screening based on PSA testing has been a matter of debate for many years, mainly because large clinical trials examining its effects on mortality have shown inconsistent results [6–13]. Indeed, a recent systematic review concluded that, at best, prostate cancer screening leads to a small reduction in disease-specific mortality over 10 years, but that it has no effect on overall mortality [14]. This is compounded by the reality that PSA screening is not without adverse consequences, such as overdiagnosis and overtreatment, with the potential for avoidable physical harm, anxiety, and costs [14–17]. Therefore, the net benefit of prostate cancer screening remains unclear, resulting in ambiguity that is reflected by different PSA testing recommendations for physicians [18–20]. Coupled with the absence of a formal screening program, this leads to uncertainty about testing for men who may otherwise be eligible for screening. Men considering screening may then receive inconsistent advice from their physicians.

In health systems predicated on evidence-based care, the attitudes and daily practice of physicians are expected to follow clinical guidelines based on the most relevant clinical trial results. Physicians should then individualize decisions according to these, also considering patient values, settings, comorbidities, general health, and other relevant characteristics. In terms of PSA testing, it has been shown that the personal beliefs and the specialization of the physician may also be relevant to the usage of PSA testing [21–23]. Variability in practice for PSA testing is not unusual among general practitioners (GPs), including those in the Netherlands. This is despite the fact that the Dutch College of General Practitioners (*Nederlands Huisartsen Genootschap* [NHG]) produced a practice guideline for Lower Urinary Tract Symptoms in Men in 2013 (henceforth, the NHG guideline) that includes a PCa screening approach due to the fact that patients frequently (wrongly) connect these two [20]. In 2014, the PSA threshold in this guideline was lowered from 4 ng/mL to 3 ng/mL to be consistent with the national guideline on prostate cancer issued by the Dutch Urological Association (*Nederlandse Vereniging voor Urologie*) [19, 24]. The guideline recommends against active offering of PSA testing to men without clinical symptoms of PCa and is actually (in 2020) under revision. Apart from being free available on the internet, the guideline is, among others, published in a Dutch scientific journal for GPs, and is part of trainings and education for GPs.

In the present study, we aimed to survey GPs in the Netherlands to assess their approaches, attitudes, and knowledge regarding the use of PSA screening for early prostate cancer detection, focusing on the prostate cancer recommendations set out in the NHG guideline.

## Methods

### Study design, setting, and participants

This cross-sectional pilot survey was performed in April and May 2016 by asking GPs in the north-east of the Netherlands to complete paper-based questionnaire. We used conventional mail to contact all GPs affiliated to the University Medical Center Groningen. In addition, the questionnaire was distributed at a training day that took place at the University Medical Center Groningen during the study period and was attended by 23 GP trainers (educational supervisors of GPs in training).

### Questionnaire development and data collection

As the questionnaire in this project was conducted in the context of a binational project, a German version of the questionnaire was translated into Dutch. Formal back and forwards translations were performed by native speakers of both German and Dutch [22]. After translation, the questionnaire was adapted to the Dutch prostate cancer guidelines and the Dutch health care system. Adaptations included for example the inclusion of digital rectal examination (DRE) results in case scenarios and questions on usage of DRE. We used the NHG guideline for reference because we expected that most GPs in the Netherlands would report using this guideline. Additional File 1 shows the Dutch questionnaire, Additional File 2 the English translation of this questionnaire.

The final iteration comprised 31 questions in five sections, addressing issues such as how and when to initiate PSA screening, the implications of results, awareness of the recommendations from national guidelines, and awareness of the results of relevant studies concerning PSA screening. Variations in daily practice of PSA testing were explored by presenting standardized case scenarios (unrelated to those listed in *Section 2.3*). In addition, some demographic and organizational data were collected about the participants and their practices.

Questions requiring graded responses were mostly answered on five-point Likert scales. To test the questionnaire on acceptance, comprehensibility, ease of use, feasibility and validity, pretests were conducted among urologists and GPs for both versions of the questionnaire. The Dutch version was regarded by a three Dutch GPs and a Dutch urologist. Based on this, the questionnaire was revised, according to the comments of the physicians. Questionnaires were completed once.

### The NHG guideline

The 2013 NHG guideline on Lower Urinary Tract Symptoms in Men provides strategies for early prostate cancer detection without advocating general PSA screening for prostate cancer [20]. Instead, guidance for PSA testing is given in two scenarios, and even then, depends on clinical assessments: [1] when an asymptomatic patient makes a request; and [2] when there is clinical suspicion.

#### Patient request (scenario 1): an asymptomatic patient requests testing

When faced with a patient request, GPs are advised to provide balanced and detailed information on the risks and benefits of screening to help the patient make an informed decision. Physicians are required to provide the following details related to prostate cancer: the risk in older men (incidence), the rarity of symptoms, and the risk of death. If the patient decides to undergo early detection, a DRE should be performed first, and a suspicious outcome should trigger direct referral to a urologist. In this scenario, a PSA test should only be ordered if the DRE is normal.

#### Suspected prostate cancer (scenario 2): the presence of suggestive symptoms or signs

When cancer is suspected based on an abnormal DRE and when patients have a life expectancy of more than 10 years they should be referred directly to a urologist without performing a PSA test. However, for those with a life expectancy of less than 10 years, the policy varies based on the suspicion of metastases. For example, a DRE should be performed when metastases are suspected, with a suspicious result triggering referral to a urologist and a normal result triggering PSA testing. The PSA test results then dictate the direction of any subsequent referral: a patient is referred to a urologist if the PSA level is ≥4 ng/mL and is referred to an oncologist if the PSA is < 4 ng/mL.

### Statistical analysis

Response proportions were calculated separately for the GPs and the GP trainers. Participant characteristics and survey responses were analyzed descriptively by absolute and relative frequencies for categorical variables. Because of the exploratory nature of the survey and the low numbers of participants within subgroups, formal statistical tests were not applied. Data analysis was done using IBM SPSS Version 25 (IBM Corp., Armonk, NY, USA).

### Ethics and data security

The German part of the study was approved by the Medical Ethics Committee of the Carl von Ossietzky University Oldenburg (No. 041/2016), which was in line with local law. For the Dutch part, no ethical approval was required, as participants were healthy volunteers and no patients, it was a one-time questionnaire, and the questions were not intrusive. Before answering the questionnaire, the participants were informed that their privacy would be respected. Data were anonymized before data handling.

## Results

### Response proportion and responder characteristics

Of the 179 postal questionnaires 65 (36%) were completed and returned. By contrast, all 23 GPs at the training day completed the questionnaires (100%). Additional File 3 shows the participation flowchart.

The characteristics of the participants and their practices are shown in Table 1. The median age of all GPs was 54 years, 25 (29%) were female, 77 (89%) had more than 10 years’ experience as a GP, and 16 (18%) worked in a practice on their own. Although almost a third (32%; *n* = 28) reported having had at least some work experience in urology during or after medical school, this was less than 1 month for most (82%; *n* = 23). More than half of the GPs (59%; *n* = 51) had attended a postgraduate training course on the usage of PSA testing.

**Table 1:** Characteristics of survey participants and practices (*n* = 88 ^a^; n (%), unless otherwise specified)

Variable	Categories	n (%)
Age (years)	n/a	54.0 (12.0) ^b^
Sex	Male	61 (70.9)
	Female	25 (29.1)
Work experience as GP	0–5 years	3 (3.4)
	6–10 years	7 (8.0)
	≥ 11 years	77 (88.5)
Amount of FTE	n/a	0.9 (0.2) ^b^
Work experience in urology	Yes	28 (32.2)
	No	59 (67.8)
Participation in a course on PSA testing	Yes	51 (58.6)
	No	36 (41.4)
Number of GPs per practice	1	16 (18.4)
	2	35 (40.2)
	≥3	36 (41.4)
Pharmacist services provided in GP practice	Yes	18 (20.7)
	No	69 (79.3)
Number of patients per practice (FTE)	n/a	2500 (650) ^b^

*FTE* Full-time equivalent, *GP* General practitioner, *IQR* Interquartile range, *n/a* Not applicable, ^a^ = numbers (%) of participants. Numbers may not add up to total, due to missing values, ^b^ = median (IQR).

### Familiarity with the NHG recommendations on prostate cancer in the LUTS guideline

Apart from 1 GP (1%), all 86 GPs had at least heard of the NHG guideline [20]. Only 3 GPs (4%) had never read or used the guideline, and of the remaining 82 (95%), half had read it and could recall the content (50%, *n* = 43). Those who reported having read the NHG guideline stated that they sought to use the recommendations in daily practice.

### Approaches and attitudes of GPs to PSA screening

GPs reported that they usually addressed the impact of PSA screening (testing in asymptomatic men) by looking at the disease-specific mortality when discussing PSA screening (Table 2). It was reported that adverse effects, such as overdiagnosis or the potential for false-positive results, were also discussed often, but that the chance of detecting prostate cancer before metastasis was rarely mentioned.

**Table 2:** Issues covered when discussing PSA screening (*n* = 88^a^, n (%))

Issue	Never	Rarely	Sometimes	Often	Always
Impact on general mortality	15 (19.7)	14 (18.4)	12 (15.8)	27 (35.5)	8 (10.5)
Impact on disease-specific mortality	9 (11.8)	9 (11.8)	14 (18.4)	32 (42.1)	12 (15.8)
Impact on chances of metastasis	26 (34.2)	21 (27.6)	13 (17.1)	12 (15.8)	4 (5.3)
Overdiagnosis	1 (1.3)	3 (3.8)	10 (12.8)	34 (43.6)	30 (38.5)
False-positive test results	2 (2.6)	4 (5.1)	6 (7.7)	33 (42.3)	33 (42.3)
Anxiety when awaiting test results	15 (19.5)	17 (22.1)	15 (19.5)	17 (22.1)	13 (16.9)
Possibility of further diagnostic tests	2 (2.6)	0 (0.0)	14 (17.9)	36 (46.2)	26 (33.3)
Possible consequences of medical policy ^b^	2 (2.6)	3 (3.9)	11 (14.3)	41 (53.2)	20 (26.0)
Referral to Thuisarts.nl (webpage) ^c^	4 (5.1)	8 (10.3)	19 (24.4)	34 (43.6)	13 (16.7)
Provide handout	24 (30.8)	23 (29.5)	18 (23.1)	8 (10.3)	5 (6.4)

*PSA* Prostate-specific antigen, ^a^ = numbers (%) of participants. Numbers may not add up to total, due to missing values, ^b^ = e.g. side effects of further diagnostics/treatment in case of positive test result, ^c^ = in the Dutch GP guideline referral to this website is recommended to help the patient to decide on PSA screening

In general, the surveyed GPs were critical of PSA screening (Table 3). Almost none would recommend testing to relatives and most of the male GPs (71%; *n* = 44) had not undergone PSA testing themselves and did not plan to do so in the future. More than 40% of the GPs (43%; *n* = 37) were not worried about missing a diagnosis of prostate cancer in patients, and in most cases, considered screening for other cancers to be more important.

**Table 3:** General attitudes of GPs toward prostate cancer screening (*n* = 88^a^, n (%))

Question	Categories	n (%)
Would you recommend the PSA test to relatives?	Definitely not	21 (25.0)
	Probably not	31 (36.9)
	Neutral	28 (33.3)
	Probably	4 (4.8)
	Definitely	0 (0.0)
Have you ever undergone a PSA test? (*men only)*	Yes	14 (22.6)
	No, but probably in the future	4 (6.5)
	No, I expect to never undergo one in the future	44 (71.0)
How concerned are you to miss prostate cancer in a patient?	Not afraid at all	3 (3.4)
	Not afraid	34 (39.1)
	Neutral	41 (47.1)
	Afraid	9 (10.3)
	Very afraid	0 (0.0)
How important do you think screening for cancer is in general?	Very unimportant	2 (2.3)
	Unimportant	5 (5.7)
	Neutral	29 (33.3)
	Important	46 (52.9)
	Very important	5 (5.7)
How important do you think screening for prostate cancer is?	Very unimportant	6 (6.9)
	Unimportant	18 (20.7)
	Neutral	38 (43.7)
	Important	25 (28.7)
	Very important	0 (0.0)

*GP* General practitioner, *PSA* Prostate-specific antigen, ^a^ = numbers (%) of participants. Numbers may not add up to total, due to missing values

### Approaches of GPs in specific case scenarios

Table 4 shows the results of the approaches of GPs when presented with different scenarios. For symptomatic patients, GPs tended to order PSA tests rarely for lower urinary tract symptoms compared with sometimes for other unclear discomfort. Over three-quarters of the GPs (76%; *n* = 67) always or often perform a DRE before requesting a PSA test. The most common indication for PSA testing was a DRE suggestive of prostate cancer (67%; *n* = 59).

**Table 4:** Approaches of GPs in specific case scenarios (*n* = 88 ^a^, n (%))

**Case scenario**	**Never**	**Rarely**	**Sometimes**	**Often**	**Always**
PSA test in case of lower urinary tract symptoms	14 (17.3)	27 (33.3)	25 (30.9)	12 (14.8)	3 (3.7)
PSA test in case of unclear discomfort	10 (11.5)	16 (18.4)	40 (46.0)	18 (20.7)	3 (3.4)
DRE before PSA test	3 (3.4)	4 (4.5)	14 (15.9)	44 (50.0)	23 (26.1)
PSA test if DRE suggestive for prostate cancer	7 (8.0)	13 (14.8)	9 (10.2)	19 (21.6)	40 (45.5)
**Case scenario**				**Yes**	**No**
Are there situations where you would not refer a patient, having a PSA level ≥ 3 ng/mL, to a urologist?				80 (94.1)	5 (5.9)
If a patient has a normal PSA level, do you check the PSA level after some time again?				17 (20.2)	67 (79.8)
Did you perform a DRE on your last patient having lower urinary tract symptoms?				70 (82.4)	15 (17.6)
**If a patient actively requests PSA screening, I will …**					
perform a PSA test without explanation				1 (1.2)	
inform the patient on the (dis) advantages of the PSA test and order it				39 (45.9)	
inform the patient on the (dis) advantages of the test and make a new appointment to decide if to order one or not				30 (35.3)	
not order a PSA test				8 (9.4)	
Others				7 (8.2)	

*DRE* Digital rectal examination, *GP* General practitioner, *PSA* Prostate-specific antigen, *n/a* Not applicable, ^a^ numbers (%) of participants. Numbers may not add up to total, due to missing values.

Almost all GPs (94%; *n* = 80) reported that there were circumstances in which they would not refer a patient with an increased PSA level to a urologist. Examples included advanced age, short life expectancy, or a plausible diagnosis of prostatitis. Some GPs used specific PSA thresholds to determine whether to refer asymptomatic patients, citing levels of 4, 5, 7, and 10 ng/mL. In patients with a normal PSA level, most GPs preferred not to retest (80%; *n* = 67).

In asymptomatic patients actively requesting PSA testing, 39 GPs (46%) said that they would agree to the request within the same session after providing information on the benefits and risks of the test. Less commonly, GPs reported they would first discuss the test but would require a separate appointment before deciding whether to perform the test (35%; *n* = 30).

## Discussion

We have presented the results of a survey conducted in the north-east of the Netherlands to assess the approaches, attitudes, and knowledge of GPs concerning the application of PSA screening for the early detection of prostate cancer. Most GPs stated that they used the NHG recommendations in daily practice when applying PSA testing in primary care: Before performing a PSA test, most stated that they discussed relevant topics with their patients, covering the many advantages and disadvantages.

Other studies on approaches to PSA testing have shown varying results. Research in the Netherlands concerning the PSA testing policy among GPs and non-urological medical specialists has revealed comparable approaches to those in the present study when faced with a patient requesting a PSA test [25]. However, before ordering a PSA test, GPs in that study performed DRE less frequently than in ours, which is consistent with the results of a survey among 303 physicians in South Africa [26]. GPs in Northern Ireland have also been shown to have similar DRE practices to those found in our study, but GPs in that study were less reserved about PSA testing (e.g., in patients with urinary tract symptoms) [27]. This finding was notable because most GPs in our survey were male, and research has indicated that male GPs are more likely to order a PSA test than female GPs [28, 29].

Another study identified considerable differences in the approaches to PSA testing between GPs in Australia and the United Kingdom (UK). Decisions about screening and PSA testing made by GPs in Australia were mostly at the discretion of individual clinicians, resulting in significant variations in practice. However, the replies of GPs in the UK reflected a clear, consistent, organizationally embedded approach based on evidenced recommendations to discourage screening [30]. The approaches reported by GPs in our survey were comparable to those reported by GPs in the UK. We agree that this suggests that health care systems, organizational structures, and guidelines collectively affect how physician’s view and handle PSA testing for early cancer detection, which is also supported by others [30]. Differences between these factors can also play a role when comparing PSA and DRE practices between countries and must therefore also be considered.

Concerning guideline adherence, a study among 55 physicians in Switzerland found that physicians generally had favorable attitudes toward clinical guidelines, but that only one-third used them very often or often [31]. Although most GPs in our study reported using the NHG guideline in daily practice, only a few followed the advice to refer a patient to a urologist without performing a PSA test if the DRE raised suspicion. After the main outcomes of the European Randomized Study of Screening for Prostate Cancer were published, the level of follow-up testing among Dutch GPs decreased after an increased PSA result [32]. The reason for this remains unclear, as do the reports by some of our respondents that they adopt their own (unsuitable) criteria and PSA cut-off values for when not to refer a patient to a urologist. In Lower Saxony, Germany, evidence has also been published showing that GPs and urologists did not treat patients in accordance with established guidelines on prostate cancer [22]. A systematic review looking at the state of PSA testing policies worldwide revealed significant variation in follow-up policies after a normal or raised PSA level, and that this is often discordant with the available practice guidelines [33]. The conflicting advice in current guidelines on prostate cancer could lead to the variations seen in daily practice [34].

There are several limitations to our survey, primarily related to the small sample and the restricted catchment area of a single university hospital, which may not have been representative of the national population. This was aggravated by the low response proportion in general and the difference in response among GPs contacted by mail compared with those surveyed at the training day. Although other postal surveys among GPs show comparable or even lower response proportions, this could be improved by relying on on-site surveys [35, 36]. The pooling of the data for the two groups is an important limitation because the conditions among those groups were different; however, we considered that the number of GPs was too small to stratify the results further. That said, we acknowledge that being a GP trainer is likely to influence opinions and knowledge on PSA testing, and that 60% of the surveyed GPs had participated in a postgraduate course on PSA testing, skewing the results to overestimate the levels of guideline adherence and knowledge. Of course, this assumes that the surveyed GP trainers involved in education and/or research are more compliant and knowledgeable than non-responders. Another factor possibly leading to an overestimation of the results may be that all answers are self-reported. However, in the Netherlands most GPs are aware of the existence and content of the Dutch GP guidelines [37]. Finally, the questionnaire was developed in German and translated to Dutch. Although we took care to ensure comparability between the two versions, we cannot exclude the possibility that the questionnaire lacks validity and reliability in the Dutch health system.

## Conclusion

Routine clinical practice regarding PSA testing in primary care seems generally to be consistent with the NHG guideline that is restrictive to PSA testing. We propose that future research should further evaluate the effects of the several PSA testing strategies.

## Supplementary Information

**Additional file 1.** Dutch questionnaire. This questionnaire was conducted among the participants of this Dutch part of the study.

**Additional file 2.** English questionnaire. English translation of the questionnaire that was conducted among the participants of this Dutch part of the study.

**Additional file 3.** Participation flowchart. GP = general practitioner. Flowchart of the invited, participated and analyzed participants of this study.

## Data Availability

Data and materials supporting the conclusion were included in the main paper. Further data were available from the corresponding author on reasonable request.

## Abbreviations

DRE: Digital rectal examination
GP: General practitioner
NHG: Dutch College of General Practitioners (*Nederlands Huisartsen Genootschap*)
PSA: Prostate-specific antigen
UK: United Kingdom
