# Supplementary material for: General practitioners’ approaches to prostate-specific antigen testing in the north-east of the Netherlands
Source: BMC Fam Pract. 2020 Dec 17;21:270. doi: 10.1186/s12875-020-01350-3 (PMC7747401; doi:10.1186/s12875-020-01350-3)
Supplement: Supplementary file 1 — Additional file 1. Dutch questionnaire. This questionnaire was conducted among the participants of this Dutch part of the study. [file 12875_2020_1350_MOESM1_ESM.docx]

Additional File 1: Dutch questionnaire

Geachte meneer, mevrouw,

Voor het project ”Vroege opsporing en behandeling van patiënten met prostaatkanker" vragen we uw medewerking. Het project wordt uitgevoerd door de afdelingen Huisartsgeneeskunde en Epidemiologie van het UMCG in samenwerking met de afdeling Urologie en Epidemiologie van het Klinikum ziekenhuis in Oldenburg.

Wij verzoeken u vriendelijk de bijgevoegde vragenlijst in te vullen, waarmee wij het gebruik van de PSA-test inventariseren. Wij behandelen uw antwoorden vertrouwelijk.

Wij stellen het op prijs als u de ingevulde vragenlijst zo snel mogelijk (het liefst binnen twee weken) terug stuurt via de bijgevoegde retourenveloppe. U hoeft geen postzegels te plakken.

Onze hartelijke dank voor uw deelname aan het onderzoek.

Voor vragen met betrekking tot dit onderzoek kunt u terecht bij:

Lisa Koops

Student stage wetenschap Geneeskunde, Rijksuniversiteit Groningen

Afdeling Epidemiologie, UMCG

Tel.: 050 361 0583

Email: l.koops@umcg.nl

Dr. Marco Blanker

Huisarts - epidemioloog, afdeling huisartsgeneeskunde, UMCG

Email: m.h.blanker@umcg.nl

Prof. Dr. Truuske de Bock

Hoogleraar oncologische epidemiologie, UMCG

Tel: 050 361 0938

Email: g.h.de.bock@umcg.nl

**Toelichting**

Deze vragenlijst is bedoeld voor huisartsen die in het (verzorgingsgebied van het) UMCG werken. Het duurt ongeveer 15 minuten om de lijst in te vullen. Er zijn geen goede of foute antwoorden mogelijk. U moet het antwoord kiezen dat het meest aansluit bij uw persoonlijke mening.

De vragenlijst bevat vragen over de volgende onderwerpen:

1. Toepassing van de PSA-test
2. Omgang met PSA resultaten
3. Richtlijnen en onderzoeken
4. Algemene vragen
5. Vragen over uzelf en uw praktijk

**Deel A: Toepassing van de PSA-test**

**Uitleg:**

*Wilt u onderstaande vragen beantwoorden vanuit uw eigen praktijkervaring?*

1. Wanneer een patiënt vraagt om vroegdiagnostiek, in dit geval een PSA-test, dan….

☐ Vraag ik een PSA-test aan zonder toelichting *(ga naar vraag 3)*

☐ Informeer ik de patiënt over de voor- en nadelen van de PSA-test en vraag ik meestal een PSA-test aan *(ga naar vraag 2)*

☐ Informeer ik de patiënt over (voor- en nadelen van de PSA-test en maken we een nieuwe afspraak om te besluiten of we de PSA-test wel of niet gaan aanvragen *(ga naar vraag 2)*

☐ Normaliter vraag ik geen PSA-test aan *(ga naar vraag 3)*

☐ Anders, namelijk ............................................. *(ga naar vraag 2)*

1. Als ik een PSA-test laat uitvoeren bespreek ik voorafgaand aan de test de volgende punten met mijn patiënt:

|  | Nooit | Zelden | Soms | Vaak | Altijd |
| --- | --- | --- | --- | --- | --- |
| Mogelijke voordelen van screening:   - Impact op algemene sterfte - Impact op ziektespecifieke sterfte - Impact op kans van metastasering | ☐ | ☐ | ☐ | ☐ | ☐ |
|  | ☐ | ☐ | ☐ | ☐ | ☐ |
|  | ☐ | ☐ | ☐ | ☐ | ☐ |
| Mogelijke nadelen van screening:   - Overdiagnostiek - Probleem fout-positieve uitslag - Mogelijke angst die ontstaat bij het afwachten van het testresultaat - Mogelijk vervolgonderzoek (vb. prostaatbiopten) bij een afwijkende uitslag van de PSA-test - Gevolgen van het beleid (daarbij meegenomen mogelijk vervolgonderzoek en eventuele behandeling bij positieve uitslag) | ☐ | ☐ | ☐ | ☐ | ☐ |
|  | ☐ | ☐ | ☐ | ☐ | ☐ |
|  | ☐ | ☐ | ☐ | ☐ | ☐ |
|  | ☐ | ☐ | ☐ | ☐ | ☐ |
|  | ☐ | ☐ | ☐ | ☐ | ☐ |
| Voorlichting:   - Na het consult verwijs ik mijn patiënt naar thuisarts.nl - Na het consult geef ik mijn patiënt informatie op papier mee | ☐ | ☐ | ☐ | ☐ | ☐ |
|  | ☐ | ☐ | ☐ | ☐ | ☐ |

1. In hoeverre bent u het met de volgende stellingen eens?

Mannelijke artsen zijn in vergelijking met vrouwelijke artsen zorgvuldiger in het uitleggen van de PSA-test.

| Helemaal niet | Niet | Neutraal | Wel | Helemaal wel |
| --- | --- | --- | --- | --- |
| ☐ | ☐ | ☐ | ☐ | ☐ |

Mannelijke artsen voeren in vergelijking met vrouwelijke artsen vaker een PSA-test uit.

| Helemaal niet | Niet | Neutraal | Wel | Helemaal wel |
| --- | --- | --- | --- | --- |
| ☐ | ☐ | ☐ | ☐ | ☐ |

Mannelijke artsen verwijzen in vergelijking met vrouwelijke artsen patiënten vaker door naar de uroloog.

| Helemaal niet | Niet | Neutraal | Wel | Helemaal wel |
| --- | --- | --- | --- | --- |
| ☐ | ☐ | ☐ | ☐ | ☐ |

1. Wanneer brengt u zelf de PSA-test ter sprake als reële optie voor diagnostiek?

|  | Nooit | Zelden | Soms | Vaak | Altijd |
| --- | --- | --- | --- | --- | --- |
| Bij mictieklachten | ☐ | ☐ | ☐ | ☐ | ☐ |
| Bij onverklaarbare klachten, zoals pijn in het bekken | ☐ | ☐ | ☐ | ☐ | ☐ |

1. Hoelang is het geleden dat u voor het laatst een PSA-test hebt aangevraagd?

........................................................................................

1. 1: Als u een PSA test aanvraagt, hoe vaak verricht u een rectaal toucher voorafgaand daaraan?

☐Nooit

☐Zelden

☐Soms

☐Vaak

☐Altijd

2: Indien een rectaal toucher verdacht is voor prostaatkanker, voert u dan nog een PSA-test uit?

☐Nooit

☐Zelden

☐Soms

☐Vaak

☐Altijd

**Deel B: Omgaan met PSA uitslagen**

1. Welk vervolgbeleid heeft u bij uw laatste asymptomatische patiënt met een onverdacht rectaal toucher en PSA-niveau van ≥3ng/mL gevoerd?

|  | Ja | Nee |
| --- | --- | --- |
| 1. De PSA na verloop van tijd gecontroleerd? 2. De patiënt rechtstreeks naar een uroloog verwezen? 3. Anders, namelijk .…………………………………………………… | ☐  ☐  ☐ | ☐  ☐  ☐ |

1. Zijn er situaties waarbij u de patiënt niet naar de uroloog verwijst bij een PSA-niveau van ≥3ng/mL?

☐ Ja ☐Nee

- - Zo ja, welke situaties zijn dit?

.................................................................................................................................................................................................................................................................................................................................................................................

1. Indien er een normale waarde van de PSA-test wordt gevonden, controleert u het PSA-niveau (op een later moment) nogmaals?

☐ Ja ☐Nee

- - Zo ja, wanneer voert u de PSA-test nogmaals uit?

......................................................................................................................................................................................................................................................

**Deel C: Richtlijnen en onderzoeken**

1. Kent u de inhoud van de volgende richtlijnen en/of resultaten uit de onderstaande onderzoeken betreffende de PSA-test? De hokjes aankruisen graag.

|  | Ja, ik ken de inhoud. | Ja, ik heb de richtlijn gelezen, maar ken de inhoud niet precies. | Ja, ik heb ervan gehoord. | Nee, ik heb er nog nooit van gehoord. |
| --- | --- | --- | --- | --- |
| 1. NHG-Standaard Mictieklachten bij mannen (Nederlands Huisartsen Genootschap) | ☐ | ☐ | ☐ | ☐ |
| 1. Multidisciplinaire richtlijn Prostaatcarcinoom (Nederlandse Vereniging voor urologie) | ☐ | ☐ | ☐ | ☐ |
| 1. Europese richtlijn voor prostaatkanker (European Association of Urology, EAU) | ☐ | ☐ | ☐ | ☐ |
| 1. European Randomised Study of Screening for Prostate Cancer (ERSPC) | ☐ | ☐ | ☐ | ☐ |

1. Gebruikt u de NHG-standaard in uw huisartsenpraktijk?

☐ Ja ☐Nee

- - Zo niet, welke richtlijn gebruikt u wel?

...........................................................................................................................

1. Welk deel van de Bijlage Prostaatcarcinoom van de NHG-standaard Mictieklachten bij mannen vindt u lastig?

............................................................................................................................................................................................................................................................................................................................................................................................................................................................................................................

**Deel D: Algemene vragen**

1. Heeft u bij de laatste patiënt met onderstaande symptomen een rectaal toucher verricht?

|  | Ja | Nee |
| --- | --- | --- |
| Patiënt met mictieklachten | ☐ | ☐ |

1. Is uw gebruik van de PSA-test veranderd sinds het verschijnen van de NHG-standaard "Mictieklachten bij mannen" in 2013?

Ik voer een PSA-test ...... uit

| Veel minder vaak | Minder vaak | Even vaak als voorheen | Vaker | Veel vaker |
| --- | --- | --- | --- | --- |
| ☐ | ☐ | ☐ | ☐ | ☐ |

1. Mannen: Heeft u ooit uw PSA-waarde laten bepalen?

☐ Ja

☐ Nee, maar in de toekomst laat ik dat waarschijnlijk wel doen

☐ Nee, ik verwacht dat ik die ook in de toekomst niet zal laten bepalen

1. Zou u naasten (uit uw privédomein) een PSA-test aanraden?

| Zeker niet | Waarschijnlijk niet | Neutraal | Waarschijnlijk wel | Zeker wel |
| --- | --- | --- | --- | --- |
| ☐ | ☐ | ☐ | ☐ | ☐ |

**Deel E: Vragen over uzelf en uw praktijk**

1. Wat is uw leeftijd? ............. jaar
2. Wat is uw geslacht?

☐Man

☐Vrouw

1. Hoelang bent u al werkzaam als huisarts?

☐ <1 ☐1-5 ☐6-10 ☐> 10

1. Hoeveel fte werkt u?

.......... fte

1. Heeft u een coschap of uw semi-arts stage bij de urologie gelopen of bent u na uw studie werkzaam geweest binnen de urologie?

☐ Ja ☐Nee

- - Zo ja, hoe lang?

☐ <1 maand

☐ 1-6 maanden

☐ 7-12 maanden

☐ 13-24 maanden

☐ > 24 maanden

1. Heeft u ooit deelgenomen aan een nascholing waarin het gebruik van de PSA-test centraal stond?

☐ Ja ☐Nee

- - Zo ja, wanneer was de laatste keer?

☐Afgelopen jaar ☐ 1-5 jaar geleden ☐ > 5 jaar geleden

1. Hoe belangrijk vindt u vroegdiagnostiek naar kanker in het algemeen?

| Erg onbelangrijk | Onbelangrijk | Neutraal | Belangrijk | Erg belangrijk |
| --- | --- | --- | --- | --- |
| ☐ | ☐ | ☐ | ☐ | ☐ |

1. Hoe belangrijk vindt u vroegdiagnostiek naar prostaatkanker?

| Erg onbelangrijk | Onbelangrijk | Neutraal | Belangrijk | Erg belangrijk |
| --- | --- | --- | --- | --- |
| ☐ | ☐ | ☐ | ☐ | ☐ |

1. Hoe bang bent u om prostaatkanker bij een patiënt te missen?

| Helemaal niet bang | Niet bang | Neutraal | Bang | Erg bang |
| --- | --- | --- | --- | --- |
| ☐ | ☐ | ☐ | ☐ | ☐ |

1. Heeft u in het verleden wel eens een prostaatkanker gemist bij een man die eerder had gevraagd om vroegdiagnostiek?

☐ Ja ☐Nee

1. In wat voor praktijkvorm werkt u?

☐Solopraktijk

☐Duopraktijk

☐Groepspraktijk

1. Bent u werkzaam in een apotheekhoudende huisartsenpraktijk?

☐ Ja ☐Nee

1. Vermeld de eerste vier cijfers van de postcode. ☐ ☐ ☐ ☐
2. Hoeveel patiënten heeft uw praktijk? *Als u het antwoord niet precies weet, probeert u dan zo goed mogelijk te schatten.*

………………………… patiënten

1. Mocht u nog opmerkingen over de vragenlijst of de PSA-test hebben, kunt u daar onderstaande ruimte voor gebruiken.

………………………………………………………………………………………………………………………………………………………………………………………………………………………………………………………………………………………………………………………………………………………………………………………………………………………………………………………………………………………………………………………………………………………………………………………………………………………………………………………………………………………………………………………………………………………………………………………………………………………………………………………………………………………………………………………………………………………………………………………………………………………………………………

U bent klaar met het onderzoek. Bedankt voor uw tijd en uw bijdrage aan deze vragenlijst!
