# Supplementary material for: General practitioners’ approaches to prostate-specific antigen testing in the north-east of the Netherlands
Source: BMC Fam Pract. 2020 Dec 17;21:270. doi: 10.1186/s12875-020-01350-3 (PMC7747401; doi:10.1186/s12875-020-01350-3)
Supplement: Supplementary file 2 — Additional file 2. English questionnaire. English translation of the questionnaire that was conducted among the participants of this Dutch part of the study. [file 12875_2020_1350_MOESM2_ESM.docx]

Additional File 2: English questionnaire

This questionnaire contains questions on the following topics:

1. Usage of the PSA test
2. Handling PSA results
3. Guidelines and studies
4. General questions
5. Questions on yourself and your practice

**Part A: Usage of the PSA test**

**Explanation:**

*Can you answer the following questions from your own practice experience?*

1. If a patient actively requests PSA screening, I will…

☐ …refer the patient for a PSA test without explanation (go to question 3)

☐ …inform the patient on the (dis)advantages of the PSA test and order it, if still requested by the patient (go to question 2)

☐ …inform the patient on the (dis)advantages of the test and make a new appointment to discuss whether the patient indeed is willing to have a PSA test (go to question 2)

☐ …in general I do not refer a patient for a PSA test (go to question 2)

☐ Other, namely (go to question 2)

1. In case I will refer a patient for a PSA test, beforehand, I will discuss the following issues with my patient:

|  | Never | Rarely | Sometimes | Often | Always |
| --- | --- | --- | --- | --- | --- |
| Potential advantages of screening:   - Impact on overall mortality | ☐ | ☐ | ☐ | ☐ | ☐ |
| - Impact on disease-specific mortality | ☐ | ☐ | ☐ | ☐ | ☐ |
| Impact on chances of metastasis | ☐ | ☐ | ☐ | ☐ | ☐ |
| Possible disadvantages of screening:   - Overdiagnosis | ☐ | ☐ | ☐ | ☐ | ☐ |
| - False-positive test results | ☐ | ☐ | ☐ | ☐ | ☐ |
| - Anxiety while awaiting test results | ☐ | ☐ | ☐ | ☐ | ☐ |
| - Further diagnostic testing in case of an abnormal PSA finding | ☐ | ☐ | ☐ | ☐ | ☐ |
| - Consequences of medical treatment (e.g. side effects of further diagnostics/treatment in case of an abnormal PSA finding) | ☐ | ☐ | ☐ | ☐ | ☐ |
| Consultation:   - After the consult I refer my patient to Thuisarts.nl (webpage) | ☐ | ☐ | ☐ | ☐ | ☐ |
| - After the consult I provide a patient information leaflet to my patient | ☐ | ☐ | ☐ | ☐ | ☐ |

1. Please state whether you agree or not with the following statements.

Compared to female physicians, male physicians are more meticulous in explaining the PSA test.

| Definitely not | Not | Neutral | Yes | Definitely yes |
| --- | --- | --- | --- | --- |
| ☐ | ☐ | ☐ | ☐ | ☐ |

Compared to female physician, male physicians conduct a PSA test more often.

| Definitely not | Not | Neutral | Yes | Definitely yes |
| --- | --- | --- | --- | --- |
| ☐ | ☐ | ☐ | ☐ | ☐ |

Compared to female physicians, male physicians refer patients more often to a urologist.

| Definitely not | Not | Neutral | Yes | Definitely yes |
| --- | --- | --- | --- | --- |
| ☐ | ☐ | ☐ | ☐ | ☐ |

1. When do you consider a PSA test as a diagnostic option:

|  | Never | Rarely | Sometimes | Often | Always |
| --- | --- | --- | --- | --- | --- |
| In case of lower urinary tract symptoms | ☐ | ☐ | ☐ | ☐ | ☐ |
| In case of unclear discomfort, e.g. pelvis pain | ☐ | ☐ | ☐ | ☐ | ☐ |

1. How long ago did you order your last PSA test?

........................................................................................

1. 1: How often do you perform a DRE before ordering a PSA test?

☐ Never

☐ Rarely

☐ Sometimes

☐ Often

☐ Always

2: In case the DRE is suspicious for prostate cancer, do you still perform a PSA test?

☐ Never

☐ Rarely

☐ Sometimes

☐ Often

☐ Always

**Part B: Handling PSA results**

1. How did you proceed in your last asymptomatic patient, having a normal DRE and a PSA level ≥3ng/mL?

|  | Yes | No |
| --- | --- | --- |
| 1. Check the PSA level after certain time? 2. Refer the patient directly to a urologist? 3. Other, namely …………………………………………………… | ☐  ☐  ☐ | ☐  ☐  ☐ |

1. Are there situations where you would not refer a patient, having a PSA level ≥3 ng/mL, to a urologist?

☐ Yes ☐ No

- - If yes, in what kind of situations?

........................................................................................................................................................................................................................................................................................................................................................................

1. If a patient has a normal PSA level, do you check the PSA level after some time again?

☐ Yes ☐ No

- - If yes, when do you check the PSA level?

....................................................................................................................................................................................

**Part C: Guidelines and studies**

1. Do you know the content of the following guidelines and/or the results from following studies on PSA testing? *Please mark with a cross where applicable.*

|  | Yes, I know the content. | Yes, I have read the guideline, but do not exactly know the content. | Yes, I have heard of this. | . No, I have never heard about this. |
| --- | --- | --- | --- | --- |
| 1. NHG guideline lower urinary tract symptoms in men (Dutch College of General Practitioners, NHG) | ☐ | ☐ | ☐ | ☐ |
| 1. Multidisciplinary guideline on prostate cancer (Dutch Urological Association, NVU) | ☐ | ☐ | ☐ | ☐ |
| 1. European guideline on prostate cancer (European Association of Urology, EAU) | ☐ | ☐ | ☐ | ☐ |
| 1. European Randomised Study of Screening for Prostate Cancer (ERSPC) | ☐ | ☐ | ☐ | ☐ |

1. Do you use the NHG guideline in your practice?

☐ Yes ☐ No

- - If not, which guideline do you use?

...........................................................................................................................

1. Which part of the prostate cancer screening approach in the Dutch GP guideline (NHG guideline) do you think is difficult?

............................................................................................................................................................................................................................................................................................................................................................................................................................................................................................................

**Part D: General questions**

1. Did you perform a DRE on your last patient having lower urinary tract symptoms?

☐ Yes ☐ No

1. Did your usage of the PSA test change since the publication of the NHG guideline lower urinary tract symptoms in men in 2013?

I conduct a PSA test ....

| Considerably less frequent | Less frequent | As frequent as before | More frequent | Considerably more frequent |
| --- | --- | --- | --- | --- |
| ☐ | ☐ | ☐ | ☐ | ☐ |

1. Men: Have you ever undergone a PSA test yourself?

☐ Yes

☐ No, but probably in the future I may undergo a PSA test myself

☐ No, I expect I will never undergo a PSA test myself

1. Would you recommend the PSA test to your relatives?

| Definitely not | Probably not | Neutral | Probably | Definitely |
| --- | --- | --- | --- | --- |
| ☐ | ☐ | ☐ | ☐ | ☐ |

**Part E: Questions on yourself and your practice**

1. What is your age? ............. years
2. What is your gender?

☐ Male

☐ Female

1. Since how many years do you work as a GP?

☐ <1

☐ 1-5

☐ 6-10

☐ >10

1. How many full time equivalent (fte) do you work?

.......... fte

1. Do you have practical experience in urology (during or after your study)?

☐ Yes ☐ No

- If yes, for how long time?

☐ <1 month

☐ 1-6 months

☐ 7-12 months

☐ 13-24 months

☐ >24 months

1. Did you ever take part in a postgraduate training course on the usage of PSA testing?

☐ Yes ☐ No

- - If yes, when was the last time?

☐ Last year ☐ 1-5 years ago ☐ >5 years ago

1. How important do you think screening for cancer is in general?

| Very unimportant | Unimportant | Neutral | Important | Very important |
| --- | --- | --- | --- | --- |
| ☐ | ☐ | ☐ | ☐ | ☐ |

1. How important do you think screening for prostate cancer is?

| Very unimportant | Unimportant | Neutral | Important | Very important |
| --- | --- | --- | --- | --- |
| ☐ | ☐ | ☐ | ☐ | ☐ |

1. How concerned are you to miss prostate cancer in a patient?

| Not afraid at all | Not afraid | Neutral | Afraid | Very afraid |
| --- | --- | --- | --- | --- |
| ☐ | ☐ | ☐ | ☐ | ☐ |

1. Have you ever missed prostate cancer in the past in a man asking you before on early detection?

☐ Yes ☐ No

1. How many GPs work in your practice?

☐ 1

☐ 2

☐ 3 or more

1. Is there a pharmacist services provided in your GP practice?

☐ Yes ☐ No

1. Please state the first four numbers of your post code. ☐ ☐ ☐ ☐
2. How many patients has your practice? *If you do not know this exactly, please try to estimate as good as possible.*

………………………… patients

1. If you have any comments on the questionnaire or the PSA test, please use the space below.

………………………………………………………………………………………………………………………………………………………………………………………………………………………………………………………………………………………………………………………………………………………………………………………………………………………………………………………………………………………………………………………………………………………………………………………………………………………………………………………………………………………………………………………………………………………………………………………………………………………………………………………………………………………………………………………………………………………………………………………………………………………………………………

Thank you for your time and your valuable contribution to this questionnaire.
