# Supplementary figures and images for: General practitioners’ approaches to prostate-specific antigen testing in the north-east of the Netherlands
Source: BMC Fam Pract. 2020 Dec 17;21:270. doi: 10.1186/s12875-020-01350-3 (PMC7747401; doi:10.1186/s12875-020-01350-3)

**Additional File 3: Participation flowchart**


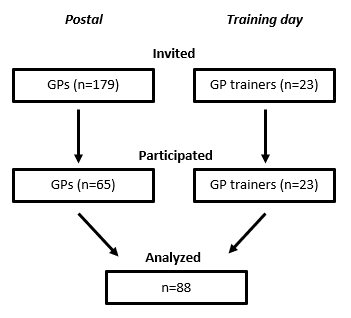


GP = general practitioner

Supplement: Supplementary file 3 — Additional file 3. Participation flowchart. GP = general practitioner. Flowchart of the invited, participated and analyzed participants of this study. [file 12875_2020_1350_MOESM3_ESM.docx]
